# Integration of substance use disorder services with primary care: health center surveys and qualitative interviews

**DOI:** 10.1186/1747-597X-9-15

**Published:** 2014-03-28

**Authors:** Darren Urada, Cheryl Teruya, Lillian Gelberg, Richard Rawson

**Affiliations:** 1 Los Angeles Integrated Substance Abuse Programs, University of California, 11075 Santa Monica Blvd Suite 200, Los Angeles, CA 90025, USA; 2 Los Angeles David Geffen School of Medicine, UCLA School of Public Health, VA Greater Los Angeles Healthcare System, University of California, UCLA Family Medicine, BOX 957087, 10880 Wilshire Blvd, Ste 1800, Los Angeles, CA 90095-7087, USA

**Keywords:** Substance use disorders, Substance abuse, Integrated care, Health policies

## Abstract

**Background:**

Each year, nearly 20 million Americans with alcohol or illicit drug dependence do not receive treatment. The Affordable Care Act and parity laws are expected to result in increased access to treatment through integration of substance use disorder (SUD) services with primary care. However, relatively little research exists on the integration of SUD services into primary care settings. Our goal was to assess SUD service integration in California primary care settings and to identify the practice and policy facilitators and barriers encountered by providers who have attempted to integrate these services.

**Methods:**

Primary survey and qualitative interview data were collected from the population of federally qualified health centers (FQHCs) in five California counties known to be engaged in SUD integration efforts was surveyed. From among the organizations that responded to the survey (78% response rate), four were purposively sampled based on their level of integration. Interviews were conducted with management, staff, and patients (n = 18) from these organizations to collect further qualitative information on the barriers and facilitators of integration.

**Results:**

Compared to mental health services, there was a trend for SUD services to be less integrated with primary care, and SUD services were rated significantly less effective. The perceived difference in effectiveness appeared to be due to provider training. Policy suggestions included expanding the SUD workforce that can bill Medicaid, allowing same-day billing of two services, facilitating easier reimbursement for medications, developing the workforce, and increasing community SUD specialty care capacity.

**Conclusions:**

Efforts to integrate SUD services with primary care face significant barriers, many of which arise at the policy level and are addressable.

## Background

Each year, nearly 20 million Americans with alcohol or illicit drug dependence do not receive treatment [1]. Moreover, patients with SUDs have health care costs that are nearly twice as high as patients without these disorders [2], contributing to the growing cost of health care. In spite of this, individuals are seldom screened for SUDs by their primary care (PC) physicians [3]. Providing SUD services in primary health care settings is feasible [3,4], can reach many more individuals than reliance on community-based specialty SUD treatment alone, promises better outcomes for patients [5-11] and can result in reduced overall health care costs ([8,12]).

Consistent with the findings above, national health care policy and practice are moving toward integrating behavioral health (mental health (MH) and SUD services) with primary healthcare [13]. The Patient Protection and Affordable Care Act (ACA) of 2010 emphasizes better coordination and integration of behavioral health and medical care [14,15] and facilitates integration by designating both MH and SUD treatment as “essential health benefits” to be covered by health plans (including Medicaid). The ACA also provides incentives for federally qualified health centers (FQHCs) to become “health homes” that specialize in the integration and coordination of care for chronic conditions, including MH and SUD [2,16]. Further, the National Drug Control Strategy calls for “a substantial expansion of substance use treatment into federally qualified health centers” [17]. Thus, FQHCs are expected to take a leading role in integration.

However, despite these steps toward integration, research to date has largely focused on the integration of MH services into PC settings while studies of SUD and PC integration have been rare [18,19]. Relatively few randomized trials have examined patient outcomes after receiving SUD services in PC settings [4,11,20-22]. Spurred in part by the passage of the ACA, however, new research is beginning to emerge. Recently Gurewich and colleagues examined patient engagement in SUD services and linking patients with SUD care in PC settings [23,24] and called for further studies. At a national level, limited FQHC survey data exist but interpreting the responses is complicated by low response rates [25].

To address the need for further information on SUD services integration efforts ahead of full ACA implementation, we conducted a small mixed methods study.

## Methods

This study consisted of both quantitative and qualitative research methods conducted in two stages. FQHCs in California were first targeted for surveys. Qualitative interviews and focus groups were then conducted among selected survey participants to gain a deeper understanding of their responses.

All participants signed informed consent forms (electronically for the web surveys). Ethical research principles were followed throughout the study, and all materials and procedures were reviewed and approved by the UCLA Institutional Review Board.

### Survey stage

#### Sample selection

The research team started sending invitations to participate in the web-based survey to a total of 18 primary care organizations, each located in one of five counties, during May 2012. All were FQHCs except one. One organization was a family practice clinic that did not have FQHC status, but was invited because it worked in parallel with FQHCs on a countywide integration project. The organization’s survey responses were similar to those of the FQHCs. More information on this project can be found elsewhere [26].Our priority was to find at least some organizations that were involved in integration efforts so we intentionally targeted counties that were known to be pursuing integration in some of their FQHCs based on presentations at UCLA’s statewide Integration Learning Collaborative^a^ and informal discussions with representatives from the County Alcohol and Drug Program Administrators Association of California. Survey data collection ended August 10, 2012.

Note this purposeful, non-random selection was not meant to produce a representative sample of the state’s FQHCs. This represents a sample of organizations that are expected to be more advanced than typical FQHCs due to the efforts in their respective counties, with the intent of finding examples that could provide lessons learned. However, these results could be interpreted as describing a likely “upper limit” with regard to current integration results that would be obtained if the study were conducted with a random sample.

Five California counties were selected for inclusion: Butte, Kern, San Francisco, Santa Clara, and Sonoma. All FQHCs located in each of the selected counties were invited to participate.The FQHCs in these counties were identified based on the list of FQHCs in the Health Resources and Services Administration’s (HRSA’s) 2010 Uniform Data Set (UDS) data (the most recent version available at the time of the survey).

Fourteen of the organizations from four counties responded (the one FQHC associated with Butte county did not respond), and one (and only one) of the FQHCs returned two separate surveys from two clinics, for a total of 15 responses. For response rate purposes we only counted one response from this FQHC, resulting in a response rate of 14/18 = 78%. Since the two clinics operate very independently, however, we included both in our analyses, for a total of 15.

#### Instrument development

The survey included questions to assess (a) the organizations’ SUD practices (screening, assessment, brief interventions, treatment, referrals, and evidence based practices), (b) the extent of integration between SUD, PC, and MH services, and (c) how services are funded, recorded (i.e. the status of electronic records), and delivered (staffing). Respondents were also asked for policy suggestions via an open ended question. The survey can be viewed at https://www.surveymonkey.com/s/fqhcsurveysample.

A single-item measure of the extent of integration was developed based on a widely used model describing levels of behavioral health and PC integration [27-29]. Based on this model, participants were asked to rate “Which of the following best describes the level of integration between __ and ___?” The blanks could be, for example, SUD and primary care services. Similar versions were used for mental health. Five levels were used:

Minimal Collaboration: SUD providers and primary care providers work in separate facilities, have separate systems, and communicate sporadically

Basic at a Distance Collaboration: Primary care and SUD providers have separate systems at separate sites, but engage in periodic communication about shared patients

Basic On-Site Collaboration: SUD and primary care providers have separate systems but share the same facility

Close Collaboration, Partly Integrated: SUD providers and primary care providers share the same facility and have some systems in common, such as scheduling appointments or medical records. Physical proximity allows for regular face-to-face communication among SUD and primary care providers.

Close Collaboration, Fully Integrated: The SUD provider and primary care provider are part of the same team. The patient experiences the SUD treatment as part of his or her regular primary care.

Respondents were these descriptions of each level and were asked to self-rate their organizations. As a validity check, a longer integration scale adapted from a mental health pilot project [30] was also included. This integration scale asked for ratings of individual aspects of integration (communication, physical and temporal proximity, services, and stigma).

#### Survey data collection

Invitations to participate were sent to the medical directors at each FQHC using both e-mail and physical addresses from UDS; they were given a two-week deadline. As the deadline approached, we sent two e-mail reminders. If the deadline passed, we repeated the process with the organization’s chief executive officer (CEO). Both the medical directors and CEOs were given the option of responding themselves or delegating the survey to an appropriate staff member who would be knowledgeable about the organization’s behavioral health practices.

The survey was hosted on a third party website (surveymonkey.com). A $100 gift card was mailed to participants upon survey completion.

#### Survey data analysis

Data were analyzed descriptively and with non-parametric inferential statistics (Wilcoxon signed-rank tests, Spearman’s rho) when analyzing levels of integration.

### Qualitative interview stage

#### Sample selection

The research team selected three organizations, one from each county participating in the survey, from among survey respondents who had indicated via the survey that they were willing to be contacted for follow-up (13 of the 15 agreed). Organizations selected based on their reports of “higher” levels of integration (e.g., basic onsite, close collaboration, partly integrated) were invited to participate in additional research activities to gain a more in-depth understanding of their integration efforts. Of the four organizations that were invited, three agreed to participate.

#### Data collection

The organizational contact (typically the person who completed the survey) identified key personnel that would be able to address the topics of interest from among administrators (e.g., Executive Director, Medical Director), staff providing direct services to patients (e.g., Physicians, Medical Assistants, Behavioral Health specialists, Psychiatrists) and patient representatives (board members or volunteers). We sent invitation letters to these potential participants.

In-person individual or group interviews with administrators and staff were conducted during August and September 2012. Every attempt was made to accommodate the schedules of the participants (e.g., when providers were not scheduled to see patients). Using interview guides, trained and experienced qualitative interviewers asked participants questions covering the following topics: patient/community needs for SUD services, SUD services provided (e.g., type, who provides), linkages to outside entities affecting the provision of or referral to SUD services, regulatory and reimbursement requirements, barriers and facilitators to integrating SUD services, and suggestions for improving the integration of services (see questions below). In addition, participants completed a brief background questionnaire. Interviews lasted 1 to 1-1/2 hours, and were voice recorded. Participants were paid $50 for focus group participation or $100 for individual interviews. All participants provided written consent to participate in the study.

### Main interview questions

#### Organizational environment (Directors only)

1. Please describe the external pressures influencing the provision of substance use disorder (SUD) services in this organization.

2. What pressures coming from within the organization have had the most influence on the provision of SUD services?

#### Provision of SUD services

3. What SUD services are provided by this organization? Please walk me through the process (e.g., who provides the services and how).

4. In your opinion, what are the barriers to providing SUD services?

5. What has facilitated the provision of SUD services?

6. What resources are needed to effectively integrate SUD services into this organization?

7. What regulatory requirements affect what and how SUD services are provided?

8. Tell me how the organization is reimbursed or paid for the SUD services that are provided.

#### Integration of SUD services with mental health and HIV/AIDS services

9. What has been or are the barriers to integrating SUD services with mental health and HIV/AIDS services?

10. What has been especially helpful in integrating SUD services with mental health and HIV/AIDS services?

#### Linkages with specialty SUD treatment providers

11. Please tell me about the nature of the linkages with outside entities affecting the provision of SUD services.

#### Other

12. Is there anything that I haven’t asked you about the provision of SUD services that you think researchers and/or policy makers should know?

#### Qualitative data analysis

Data analysis was conducted on externally transcribed audio recordings of the interviews. Transcripts were reviewed against the audio recordings for accuracy and completeness, edited, and uploaded into Atlas.ti, a computerized qualitative data analysis software program, for coding. Analyses were conducted simultaneously with data collection and data interpretation in an iterative process according to established and accepted procedures for qualitative research [31-33]. Analyses were conducted simultaneously with data collection and data interpretation in an iterative process and approached with grounded theory [32]. The procedures involved involved the repeated reading of the transcripts, development of a code list, coding the data to identify emerging patterns relevant to the study objectives and utilization of the constant comparative method [32]. Development of the preliminary code list was guided by the interview topics (e.g., SUD services, barriers to integration, external pressures). Inductive codes that emerged from the data were added (e.g., reimbursement), and code lists were adjusted and refined, including primary and secondary codes. The major thematic categories for barriers and facilitators to retention emerged from the data. Relevant data from the interviews were cross-checked against the survey responses by comparing responses to similar questions in both the survey and interview guide (e.g., SUD services provided on site, SUD practices in use, barriers and facilitators) for each FQHC, and discrepancies and other contextual data (e.g., position of the survey respondent and interviewee, whether the survey was filled out according to the general practices of the organization across sites or answered with the largest primary care site in mind) were reviewed and noted.

## Results

### Interview participant characteristics

The sample is comprised of 18 participants (four to seven participants at each site), which included staff who reported their primary job as director or medical director (five), case manager (two), physician (two), physician assistant (one), psychologist (one), behavioral health clinician (two), SUD counselor (one), medical social worker (one), and patient representatives (three), Half of the sample was male; 67% identified as White, 22% as Hispanic. Participants’ average age was 44.94 years (*s.d.* 10.77) and their average length of employment at the organization was 66.27 months (*s.d.* 61.83).

The nine major themes that emerged from the survey questionnaire results and qualitative interview finding include: integration measure, integration and rating of SUD services, SUD screening, SUD counseling, evidence-based treatment practices, reimbursement for SUD services, SUD training, barriers to integration, and facilitators of integration. Perspectives of participants collected from the qualitative interviews are provided, where relevant, to illustrate the quantitative survey results.

### Integration measure

Scores from the single-item level of integration measure were highly correlated with the longer multi-dimensional integration scale (*ρ* = .85, p < .001), suggesting good convergent validity.

### Integration and rating of SUD services

#### Survey findings

Even though we targeted “high integration” counties for the surveys, seven (50%) of organizations described their SUD services as having minimal or only “basic at a distance” collaboration with PC, while the other seven (50%) described it as “Close Collaboration, Partly Integrated” (i.e., four on a five-point scale). By comparison, for MH and PC integration, eleven (79%) reported close or full integration. Thus there was a non-significant trend toward SUD services being less integrated than MH services based on a Wilcoxon signed rank test of the ratings (*Z* = 1.54, *p* = .13).

Respondents also rated their organization’s effectiveness in treating SUD problems on a five-point scale ranging from not at all effective to extremely effective and had significantly lower ratings for effectiveness in treating SUD ($\bar{x}$ =2.86, *s.d.* = 0.95) than MH disorders ($\bar{x}$ =3.57, *s.d.* = 1.09) (*Z* = -2.06, *p* < .05). The lower effectiveness for SUD treatment did not appear to be due to differences in attitudes toward treating SUD, however. Participants indicated that it was equally possible to treat SUD and MH effectively ($\bar{x}$ =4.21, *s.d.* = 0.70 and mean = 4.14, *s.d.* = 0.53 respectively on a five point scale, Z = -0.38, *p* = .71). Instead the difference in SUD and MH treatment effectiveness appeared to reflect training. Participants indicated significantly lower knowledge of SUD treatment EBPs than MH EBPs ($\bar{x}=3.29,s.d.=1.14\;\mathrm{vs}\;\bar{x}=3.93,s.d.=0.83$), *Z* = -1.98, p < .05). The same results were found using dependent t-tests or Wicoxon signed rank tests.

Physical and temporal separation in services was common. In only two cases (13%) were PC and SUD services located in the same building. When referrals for SUD services were made, ten (64%) indicated that a delay of more than seven days was typical.

#### Interview findings

While the nature and level of integration of SUD services varied among the three organizations, all of the sites used patient care team models, which included providers who address SUD issues or have access to specialists onsite or offsite who can provide consultation, therapy, or referrals for specialized SUD services. Providers indicated that SUDs may be one of a number of interconnected issues that their patients may be dealing with, thus having a multi-disciplinary staff was essential.

“I can tell you that we don’t have anybody that doesn’t need an integrated treatment team. Very few people that come through here that don’t need multiple services…Because we’re all here together, it really does improve access”.

### Screning practices

#### Survey findings

Eight of the 15 responding (53%) organizations reported screening all patients for SUD; three (20%) reported screening a targeted group of patients; and four (27%) did not screen for SUD.

#### Interview findings

Participants provided insight into the screening process. While some providers used formal standardized instruments, in other cases “screening” was a more informal process. For example, one of the PC providers explained:

“I ask a lot of question about drugs and alcohol. ‘Do you drink alcohol sometimes? What do you like to drink? What’s it like? How’s that going for you?’ Just really try to quantify it”.

### Psychosocial counseling

#### Survey findings

Twelve (86%) of 14 respondents reported having individual SUD counseling available onsite, and four (27%) reported having group counseling onsite.

#### Interview findings

The degree to which the available counseling could be considered “SUD treatment” varied. As one provider described it:

“*Of course you don’t ignore somebody’s substance abuse, particularly if it’s impacting them, but it’s not substance abuse counseling. That’s something more specialized”.*

### Evidence based practices

#### Survey findings

While we were unable to measure fidelity via the survey, the evidence based practices (EBPs) that providers most commonly indicated they used (selected from a list) were motivational interviewing, cognitive behavioral therapy, and social skills building, each reportedly in routine use by seven of the 14 survey respondents (50%). Use of another EBP, medication assisted treatment, is infrequent. Only three (21%) reported prescribing buprenorphine (brand names: Subutex, Suboxone) “sometimes” and none do it routinely. Only one (7%) reported prescribing injectable naltrexone.

#### Interview findings

Providers at all three of the organizations mentioned that they used motivational interviewing, although according to one participant, providers may not necessarily focus this practice on SUD issues.

With respect to SUD medication assisted treatment, two of the sites had physicians who prescribed buprenorphine, and both offered group visits for patients prescribed buprenorphine. At one of the programs, a multi-disciplinary care team (physician, medical social worker, psychologist, psychiatrist) conducted these group visits to provide monitoring, education, dose adjustments, service linkage, and peer support. Providers indicated that this format was effective and efficient in terms of patient access to and provider delivery of care.

### Reimbursement for SUD services

#### Survey findings

Nine of the FQHCs (64%) included SUDs in their FQHC prospective payment system (PPS) rate. Other revenue sources reported included billing to other county health sources (four, 29%), paying for services through grant funding (five, 36%), and services provided without any billing (three, 21%). Six out of the seven organizations that reported close collaboration also reported that they found ways to fund their SUD services through use of the PPS rate, suggesting the importance of reimbursement for integration efforts.

#### Interview findings

Participants at all three sites indicated that reimbursement for SUD services is a challenge, even among programs that receive Ryan White Act funds, which include SUD services, or grants to provide health care for patients who are homeless.

### Training

#### Survey findings

Twelve of the fourteen respondents (86%) agreed that additional SUD-related training would be helpful for their clinic staff.

#### Interview findings

Consistent with the survey finding, several participants indicated during the interviews that they had an interest in or had recently participated in SUD training.

### Barriers to integration

Additional qualitative data were gathered on barriers and challenges to SUD service provision and integration. The main themes that emerged are presented below.

#### Workforce training

According to several participants at two of the sites, it is challenging to find behavioral health providers who have an interest in and the skill set (e.g., flexibility, bilingual Spanish and English) to work in community health care settings. For example, such specialists may be more comfortable with a traditional approach that is focused on long-term counseling and where patients are seen by appointment for a particular period of time (50 minutes) rather than a crisis intervention or short-term approach that addresses the immediate needs of patients.

“The way therapists are trained is not conducive to what’s needed in a community health center. It doesn’t work. The 50 minute hour is useless in a community health center, I think, but that’s [how] therapists are trained… To me, there’s a new breed of therapists that we need that I’ve only met one or two that fit that role over the years”.

In addition, some of the participants interviewed commented that providers find if challenging to deal with patients with chronic pain and/or SUDs. Providers vary widely in their willingness to work with these patients, as the following comment exemplifies.

“The chronic pain stuff and substance abuse…A lot of docs come into it with biases that are not particularly helpful. Some of them aren’t all that amenable to change”.

#### Billing for same-day SUD services

Providers are not reimbursed through Medi-Cal (California’s Medicaid system) for physical and MH services delivered on the same day. Patients must return on another day or providers either are not paid for services they actually provide or must develop “workarounds” to deliver the needed behavioral health services. In theory, FQHCs can account for same day visits by including them in their PPS rate, but this is a long and costly process that FQHCs have been reluctant to undertake, often absorbing the costs instead.

“A huge issue that we’ve struggled with along with everybody else is that you can’t bill for more than one provider on a single day. So here we have these group medical visits where the psychiatrists, the primary care physician, the psychologist and a clinical social worker are all seeing the patient on the same day. So basically we’ve had to eat that cost largely”.

#### Providers eligible to bill

Another billing issue raised across the sites is that SUD services provided by Marriage and Family Therapists (MFTs) and certified SUD counselors currently cannot be billed to Medi-Cal.

“That’s the number one [barrier]. I mean, we would have substance abuse counselors on staff if you could bill for it, but you can’t bill for it…The FQHCs, that’s basically how they run, is on billable services”.

#### Barriers to SUD medication reimbursement

The process for obtaining prior approval for non-formulary medication to treat opioid addiction (e.g.,buprenorphine, injectable naltrexone) can be complicated. According to several participants at one of the programs with multiple providers licensed to prescribe buprenorphine, although a provider can submit a treatment authorization request (TAR) and receive approval from Medi-Cal for the medication, the paperwork could be complicated and there may be delays in receiving such approval, which may negatively impact patients (e.g., dosing, dose adjustments) and providers trying to deliver care (e.g., time and effort to submit and follow-up with paperwork).

“A lot of our patients at some point end up getting on either Medi-Cal or Medicare…Things become a lot more complicated at that point, where there’s this constant fight with the insurance company requesting approval for this non-formulary medicine, to the extent that…it puts the treatment of our patients at risk, ‘cause they are not able to get the medicine”.

#### SUD resources in the community for patient referrals

Participants interviewed in all three of the selected counties commented that there are very limited or no community resources/services for SUDs, particularly residential treatment and detoxification. The following quotations from providers exemplify this theme.

“Outpatient is something, but it’s really not addressing the level of need that my patients have. It’s like putting someone with an active GI bleed in-I don’t know-an observation bed or something. These are ICU-level addicts, and…we’re offering them outpatient at best. A lot of the times, we don’t even offer ‘em that because they try to call for inpatient for three weeks. They get nowhere. They just end up leaving and giving up”.

### Facilitators of integration

Additional qualitative data were collected on facilitators to SUD service provision and integration. The main themes that emerged were PC provider access to SUD expertise and finding providers with the right “fit”.

#### PC providers access to SUD expertise

Participants in all three organizations expressed that PC providers feel more willing, and able to deal with SUD issues when there are behavioral health specialists and other providers to provide SUD consultation and services. According to providers interviewed, multi-disciplinary teams are able to more effectively address patients’ many and diverse needs, and may also decrease the burden on any one provider.

“They [primary care providers] really value having [the SUD counselor] there. They can talk about substance abuse issues, but to have someone to do the kind of follow-up… The doctors love to have someone who can specifically address that so that they can get them to a place where they can prescribe”.

#### Providers with the right “fit”

Participants across the three sites similarly commented about the importance of having the right staff (e.g., skills, personality, training, flexibility, interest in and comfort level with addiction) when trying to integrate SUD services into PC settings. Many of the patients in FQHCs need care in multiple areas, including the harmful use of alcohol and drugs.

“You really need a staff who actually are okay working with drug users. You know, who don't think that they're weak, bad people, don't think that they shouldn't be wasting their time on them anyway”.

## Discussion

Based on the survey and interview results, SUD services were not well integrated into PC settings even in counties that were selected based on their integration efforts. There was a trend for SUD services to be not as well integrated as MH services were, and even when activities such as screening and counseling were reported, interviews showed that these activities were sometimes informal and unspecialized. While the implementation of SUD and PC integration must take place at the clinic and provider levels, many of the barriers cited must also be addressed at the policy level. When participants were asked for their thoughts on policy issues in the survey and interviews, several concrete policy suggestions recurred across participants:

• **Expand the SUD workforce that can bill Medi-Cal in FQHCs.** In particular, FQHCs want to add Marriage and Family Therapists (MFTs) to the list of staff that can bill. Currently only LCSWs and psychologists can bill for behavioral health. This would require legislation to amend the state’s Welfare and Institutions Code, relating to Medi-Cal, and a change to the state plan.

• **Enable same-day billing of two services.** This is consistent with best practices regarding “warm hand-offs” between primary care and SUD or MH. If a patient must make an appointment to come in another day to receive these services, they will often become “no shows” and an opportunity to address their problems will be lost. In theory, FQHCs can incorporate this into their PPS rate, but the process is time consuming that requires long periods before FQHCs can recoup costs, causing “major frustration” [34], and therefore a disincentive for FQHCs to pursue it.

• **Improve access to medications that have been shown to be effective in treating opioid addiction.** Although providers may submit a treatment authorization request for Suboxone, providers and support staff report finding the process to be time consuming, labor intensive, and frustrating, which can negatively impact patient care and providers’ willingness or ability to provide such medications.

• **Develop the primary care workforce to effectively provide SUD care.** SUD and recovery should be covered in the curriculum for medical and nursing students, social workers, psychologists, and other direct care providers so they are comfortable and effective in addressing patients’ alcohol and drug use.

• **Increase capacity in the community for specialized SUD and support services, especially residential treatment and detoxification.** Patients who are referred out by providers in FQHCs for specialty SUD services find it extremely difficult to access needed services. California does plan to provide an “enhanced” Medi-Cal benefit for SUD treatment services in 2014 [35], which may help to improve access to specialty services in the community. A better option for some patients might be have FQHC staff provide these services on-site, but this enhanced benefit is mostly provided through a Drug Medi-Cal carve out that can only be billed by certified specialty Drug Medi-Cal providers, not FQHC staff.

A major barrier to these suggested changes appears to be the perception that all of them may add Medi-Cal (and thus) State general fund costs. For example in 2012 a bill that would have added MFTs as reimbursable providers in California passed 17–0 out of the Assembly Health Committee, but was not passed by the Assembly Appropriations Committee. In fact, research and the real-world experience of insurance companies is that treating SUD problems often leads to savings in total medical costs [8,12]. SUD treatment also produces savings in non-medical areas such as incarceration [36].

Ultimately the solution to many of these problems may be payment reform that results in payments to organizations on a per-member basis, which will incentivize cost savings while potentially removing many of the barriers discussed above (billable staff, same-day service billing, medication billing). Although FQHCs primarily operate under the PPS model today, the ACA encourages such capitated and other shared savings models through the formation of patient centered health homes and accountable care organizations. Future policy and research efforts are needed to ensure that these models live up to their potential to facilitate behavioral health and primary care integration. FQHCs and policymakers can also look to models of integration successfully used by the Veterans Administration [37-39] for additional lessons learned.

### Study limitations

This study had several limitations. Findings are drawn from a non-random sample who were willing to participate in the surveys and interviews and who were available when the site visits were conducted. Thus, their perspectives may not represent the perceptions and experiences of staff who were not interviewed or organizations that were not selected. They do, however, provide insight on what organizations that are integrated, albeit to various extents, are actually doing in terms of practices and SUD service provision and the facilitators of and barriers to such integration. Our interview and survey samples are also relatively small. The lack of significant relationships between some variables may be in part due to the small size of the study, and should not be interpreted as evidence for the absence of such relationships. The small size and purposeful sampling may limit the generalizability of the policy suggestions.

## Conclusion

Efforts to integrate SUD services with primary care face significant barriers, many of which arise at the state policy level and are addressable through the policy suggestions provided. Expanding and developing the workforce, enabling same-day services, improving access to medications, and improving access to specialty care should be priorities according to FQHC interviewees that are currently involved in integration efforts. Next steps include discussing these findings with state and county policymakers with the goal of exploring these policy changes.

## Endnote

^a^The Integration Learning Collaborative takes the form of monthly conference calls and quarterly in-person meetings in connection with meetings of the Department of Alcohol & Drug Programs/County Alchohol and Drug Program Administrators Association of California. All counties are required by law to participate in these meetings. As a result nearly all of the 58 counties were represented at each of the in-person meetings. For more information see http://www.uclaisap.org/affordable-care-act/html/learning-collaborative/index.html

## Competing interests

The authors declare that they have no competing interests.

## Authors’ contributions

DU led the conception and design of the study, survey data collection and analysis, and writing and revision of the manuscript. CT led the qualitative design, data collection, and analysis and contributed to the writing and revision of the manuscript. LG and RR contributed to the study conception and design and provided revisions to the manuscript on important intellectual content. All authors read and approved the final manuscript.

## Authors’ information

DU, CT, and RR are researchers at the UCLA Integrated Substance Abuse Programs (UCLA ISAP). RR is the director of UCLA ISAP. The three of them are involved in evaluation, training, and technical assistance efforts related to the integration of SUD services with primary care. LG is Professor in the Department of Family Medicine and School of Public Health at UCLA and the VA Greater Los Angeles Healthcare System. She conducts community-based research on the health, access to care, and quality of care of vulnerable populations.
